# Comparing Virtual Reality–Based and Traditional Physical Objective Structured Clinical Examination (OSCE) Stations for Clinical Competency Assessments: Randomized Controlled Trial

**DOI:** 10.2196/55066

**Published:** 2025-01-10

**Authors:** Tobias Mühling, Verena Schreiner, Marc Appel, Tobias Leutritz, Sarah König

**Affiliations:** 1 Institute of Medical Teaching and Medical Education Research University Hospital Würzburg Würzburg Germany

**Keywords:** virtual reality, VR, objective structured clinical examination, OSCE, medical education, technological proficiency, assessment, clinical competence, item characteristics, discrimination power, acceptance, technical feasibility, effectiveness, comparative study, physical stations, medical students, standardized patients, cost-effectiveness

## Abstract

**Background:**

Objective structured clinical examinations (OSCEs) are a widely recognized and accepted method to assess clinical competencies but are often resource-intensive.

**Objective:**

This study aimed to evaluate the feasibility and effectiveness of a virtual reality (VR)–based station (VRS) compared with a traditional physical station (PHS) in an already established curricular OSCE.

**Methods:**

Fifth-year medical students participated in an OSCE consisting of 10 stations. One of the stations, emergency medicine, was offered in 2 modalities: VRS and PHS. Students were randomly assigned to 1 of the 2 modalities. We used 2 distinct scenarios to prevent content leakage among participants. Student performance and item characteristics were analyzed, comparing the VRS with PHS as well as with 5 other case-based stations. Student perceptions of the VRS were collected through a quantitative and qualitative postexamination online survey, which included a 5-point Likert scale ranging from 1 (minimum) to 5 (maximum), to evaluate the acceptance and usability of the VR system. Organizational and technical feasibility as well as cost-effectiveness were also evaluated.

**Results:**

Following randomization and exclusions of invalid data sets, 57 and 66 participants were assessed for the VRS and PHS, respectively. The feasibility evaluation demonstrated smooth implementation of both VR scenarios (septic and anaphylactic shock) with 93% (53/57) of students using the VR technology without issues. The difficulty levels of the VRS scenarios (septic shock: *P*=.67; anaphylactic shock: *P=*.58) were comparable to the average difficulty of all stations (*P*=.68) and fell within the reference range (0.4-0.8). In contrast, VRS demonstrated above-average values for item discrimination (septic shock: r'=0.40; anaphylactic shock: r'=0.33; overall r'=0.30; with values >0.3 considered good) and discrimination index (septic shock: D=0.25; anaphylactic shock: D=0.26; overall D=0.16, with 0.2-0.3 considered mediocre and <0.2 considered poor). Apart from some hesitancy toward its broader application in future practical assessments (mean 3.07, SD 1.37 for VRS vs mean 3.65, SD 1.18 for PHS; *P*=.03), there were no other differences in perceptions between VRS and PHS. Thematic analysis highlighted the realistic portrayal of medical emergencies and fair assessment conditions provided by the VRS. Regarding cost-effectiveness, initial development of the VRS can be offset by long-term savings in recurring expenses like standardized patients and consumables.

**Conclusions:**

Integration of the VRS into the current OSCE framework proved feasible both technically and organizationally, even within the strict constraints of short examination phases and schedules. The VRS was accepted and positively received by students across various levels of technological proficiency, including those with no prior VR experience. Notably, the VRS demonstrated comparable or even superior item characteristics, particularly in terms of discrimination power. Although challenges remain, such as technical reliability and some acceptance concerns, VR remains promising in applications of clinical competence assessment.

## Introduction

Objective structured clinical examinations (OSCEs), first described in 1975 [1], have long since been recognized and accepted as a reliable, valid, and objective method to assess clinical competencies in medical education. They are organized in a circuit format and use stations featuring standardized cases and predefined assessment criteria using checklists or global rating scales to objectify the evaluation by the assessor. By breaking down the clinical tasks into multiple subtests, various skills aligned with learning objectives can be evaluated simultaneously [2]. Presently, this examination format is administered using either standardized patients (SPs) or simulators. SPs are trained actors who are provided with specific cases and then present themselves as patients to students [3]. Students using this method can exhibit their proficiency at the third level, “shows how,” of the competency framework by Miller [4]. This surpasses traditional formats such as oral or written examinations, which generally focus on Miller’s second competency level, “knows how,” linked to the application of knowledge. Nevertheless, OSCEs are subject to a number of significant limitations, with one of the primary issues being their resource-intensive nature, both in terms of materials and personnel [2]. The time-intensive training and deployment of SPs also cause significant financial expense. In addition, in terms of content, OSCEs may not comprehensively capture the complexity of emergency scenarios. Using SPs often proves inadequate at accurately simulating the complex pathophysiology found in living organisms. In particular, healthy actors may struggle to depict diseases in all their nuances, and invasive procedures cannot be executed.

Virtual reality (VR) simulation as a supplementary method in medical education has received a high degree of acceptance among learners and shown promising results in terms of learning outcomes [5,6]. Importantly, the use of VR-based scenarios in assessments may potentially overcome the aforementioned limitations: Virtual patients can display symptoms and findings that realistically represent illnesses, and a computed dynamic physiology can replicate appropriate responses to medication or invasive interventions such as endotracheal intubation or administration of catecholamines. Additionally, digitally assisted assessment offers the possibility of relieving examiners through automated and objective recording of the results. Considering these advantages, using VR scenarios of medical emergencies in OSCE stations appears promising, but evidence relating to technical reliability and cost-effectiveness remains limited [7]. Indeed, to ensure smooth examinations, a sufficient level of hardware and software maturity is essential to avoid interruptions resulting from technical issues. Furthermore, the technically available scenarios must align with the learning and assessment objectives of the respective curriculum.

Given these prerequisites, application examples in this domain are limited. A pilot study did showcase the successful use of VR-based training of cardiopulmonary resuscitation in an examination setting [8]. Another study reported that skills assessment using 360° videos delivered via VR head-mounted displays can yield valid outcomes [9]. We also introduced a VR-based simulation training course on complex medical emergencies into the curriculum at our institution in 2020 [10]. It has been continuously refined to meet the aforementioned technical requirements and is now a suitable candidate for use as an examination tool.

Although VR holds promise for medical assessment, research into large-scale OSCEs and technical feasibility is limited. Additionally, only a few studies address the didactic requirements, such as test quality and consistency of results. In this study, we aimed to determine whether the theoretical benefits of VR are realized within the tight schedule of an already established routine OSCE in the curriculum with a full cohort of students.

On this basis, we aimed to address the following: (1) whether it is both organizationally and technically feasible to integrate VR-based stations focused on emergencies within an existing curricular OSCE framework, (2) whether VR-based stations display item characteristics (such as item difficulty, item discrimination, discrimination index) comparable to their physical counterparts that test identical content, and (3) how students perceive and to what extent they accept the VR-based stations.

## Methods

### VR-Based Simulation Training

STEP-VR (version 0.13b; ThreeDee GmbH) was used as the VR-based simulation of complex emergencies together with the hardware setup and head-mounted displays essentially as described previously [10].

### Study Design

**Figure 1:**
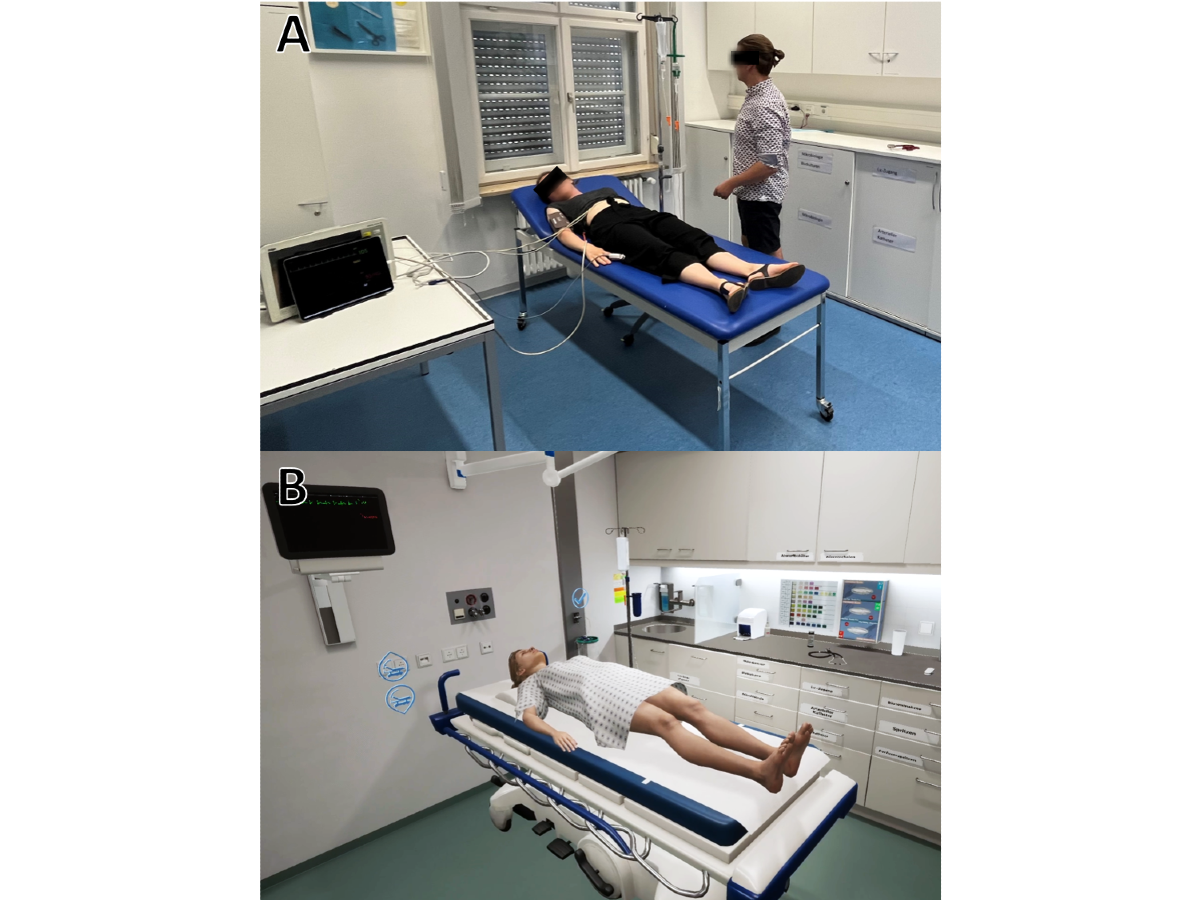
Representative scenes from the (A) virtual reality–based station (VRS) and (B) the physical station (PHS) counterpart.

The study was conducted at a medical school in Germany (University of Würzburg) at the end of the fifth year of study toward the degree of medicine. The already established curricular OSCE was designed traditionally as a circuit with 10 stations, with 2 circuits running parallel to each other to increase throughput and reduce the total examination duration to 2 days. Of the 10 stations, 5 were case-based focusing on a number of specialties, and 4 were skills-based. Central to the study was the tenth station, the medical emergencies station. This station was the only one available in 2 separate modes: either a VR-based or a real-world mode, which we designated as a VR-based station (VRS) or physical station (PHS), respectively. The VRS and PHS were designed to be visually and functionally as similar as possible, including the case description and task assignment for the students, case dynamics during simulation (expressed through changes in vital parameters), and functionality of the emergency room environment (Figure 1). At each station, students were given 1 minute to read the case description and task, followed by 9 minutes to complete the examination. To mitigate the consequences of students potentially sharing information, scenarios in all stations were switched at differing intervals.

Throughout the semester, students received comprehensive preparation for the OSCE, with particular emphasis on its implementation of emergencies in VR. They were given a script detailing the acute treatment of different types of shock. Additionally, a tutorial video was made available to familiarize students with the VR equipment and software. All students participated in STEP-VR as part of mandatory 3-hour small group sessions. However, during these sessions not all students were active in the VR; some were observing through a screen displaying the first-person perspective. Furthermore, each student had the opportunity to practice using the system beforehand in a voluntary training session.

In the VRS and PHS on day 1, students encountered a patient with an initial diagnosis of fistulizing Crohn disease leading to septic shock. The assessment focused on the managing measures of the “One Hour Bundle” (ie, actions to treat sepsis to be executed within 1 hour) and the decision-making process for either interventional or surgical abscess drainage. On day 2, students faced the challenge of stabilizing a patient experiencing anaphylactic shock triggered by the painkiller metamizole. In addition to managing the associated respiratory distress, they were required to advise the patient on measures to prevent recurrence. All medical content was based on established guidelines and reviewed by experienced faculty members. At the outset of the OSCE, participants were randomly assigned to 1 of the 2 parallel and simultaneous circuits. Ultimately, each student had to undertake 1 of the 2 scenarios either within the VRS or the PHS. A backup VR setup was always on hand to address any technical issues.

In both the VRS and PHS, students were provided with all the essential information for the case in the task description (as a sign on the door prior to entering the station). This encompassed medical history, physical examination, and diagnostic test results. The subject and goal of the examination were focused on acute treatment (taking immediate actions) and making decisions for the next steps (correct indication for intervention or surgery or providing recommendations). Students were provided with assistance when donning the head-mounted display and controllers. The first-person perspective of the students was transmitted to a screen, allowing assessors to view the students’ actions. The performance was rated with standardized candidate assessment forms.

For students assigned to the VRS but who chose not to use VR technology owing to reservations (such as past instances of simulation sickness), a tutor took over the operation of the headset and controllers. The student could observe the scenario on the screen and had to guide the tutor with the appropriate actions and measures through verbal commands. However, data from these students were not included in the study.

The 5 case-based stations (internal medicine, surgery, family medicine, pediatrics, gynecology) were comparable to the VRS and PHS. In the case-based stations, the scenarios were changed every half day, so that 4 different scenarios were used for each specialty. These also assessed students’ management and clinical decision-making, especially with regard to diagnostic and therapeutic measures. The other 4 stations, which focused on procedural competence in highly standardized scenarios (eg, postoperative blood transfusion), were incomparable with the other stations and thus excluded from subsequent analysis in this study.

### Randomization

Randomization was performed using computer-generated random numbers. The block size was set to 18 for each session to divide participants into 2 different circuits (including VRS and PHS). The randomization sequence was created prior to the study, and all OSCE stations were conducted within the randomized group allocation.

### Blinding

VRS and PHS were substantially different, so that both participants and assessors were aware of the group allocation. Thus, blinding was not possible in this study.

### Sample Size Calculation

As the number of participants was constrained by the total number of students available in the semester, sample size was not determined through a specific power analysis. Instead, a full census was conducted, including all available students at the end of their fifth academic year.

### Feasibility Evaluation

Throughout the implementation process, various measures of organizational and technical feasibility [11] were evaluated. Building on extensive data from our group and others on the integration of VR scenarios in medical training, along with promising results from pilot studies on partially immersive VR-based assessments (eg, using 360° videos, as mentioned in the Introduction section), we opted to trial the approach with an entire cohort of students. Feasibility outcomes were documented across key areas, including acceptability, demand, implementation, practicality, adaptation, integration, expansion, and limited efficacy. Additionally, a cost comparison between the VRS and PHS modalities was conducted.

### Collection of Performance and Evaluation Data

#### Assessment of Student Performance

To assess students in both the VRS and PHS as equally as possible, identical candidate assessment forms were used. They consisted of 10 items (septic shock) and 13 items (anaphylactic shock) from the categories of (1) additional monitoring and diagnostics, (2) treatment and definitive diagnosis, and (3) subsequent actions and advice. Scoring for each item was either binary (criteria met or not met, corresponding to 1 or 0 points) or ternary (criteria fully met, partially met, or not met, corresponding to 2, 1, or 0 points). When calculating the total sum, all items were equally weighted and normalized to a maximum of 1 point. The candidate assessment forms are outlined in Tables S1 and S2 in Multimedia Appendix 1.

#### Online Survey for Evaluation and Feedback From Students

Right after the OSCE, students were invited to participate in an online survey accessible via a QR code to be scanned and performed following the examination. The survey encompassed demographic parameters such as age and prior experience with VR technology. It also featured a total of 18 items divided into various topics: stress experience (3 items), usability (2 items), preparation (2 items), acceptance (5 items), subjective performance (3 items), and general rating (3 items to share views on the OSCE’s value, its personal relevance, and its future inclusion). Items were rated on a 5-point Likert scale from “strongly agree” (5 points) to “strongly disagree” (1 point). Students could also provide qualitative feedback via 2 open-ended questions.

### Analysis of Item Characteristics

The OSCE’s item characteristics for both the VRS and PHS across the 2 scenarios as well as for the other 5 case-based stations, each comprising 4 distinct scenarios, were detailed. Parameters, such as difficulty P (average participant score at the station), discrimination r’ (correlation of station scores with overall scores excluding that station), and discrimination index D (difference in difficulty between high and low performers based on the top and bottom 33rd percentiles) were computed for all OSCE stations, as suggested by Möltner et al [12]. Regarding item difficulty (P), values between 0.4 and 0.8 were considered ideal [12]. For item discrimination (r'), values above 0.3 were considered good, values between 0.2 and 0.3 were considered acceptable, and values below 0.2 were considered insufficient [12]. For the discrimination index (D), values above 0.4 were considered excellent, values between 0.3 and 0.4 were considered good, values between 0.2 and 0.3 were considered mediocre, and values below 0.2 were considered insufficient [12].

The computation of item statistics was carried out using R 4.3.1 [13]. For the sake of clear presentation, the average was provided for each of the other 5 case-based stations. Reporting was primarily conducted in accordance with the CONSORT-EHEALTH recommendations [14]. The corresponding checklist is provided in Multimedia Appendix 2.

### Analysis of Survey Data

Student responses from the VRS and PHS were analyzed using descriptive statistics such as counts (both absolute and percentages), means, and SDs. Nominally scaled variables were compared using the chi-square test. For continuous survey data, such as aggregated Likert data, the Shapiro-Wilk test was initially used to assess normality. Since the assumption of normality was violated for most items, the Wilcoxon rank-sum test was used for comparisons between the groups (VRS vs PHS).

A “thematic analysis” approach was used to summarize the anonymous open-ended responses from VRS participants, following the method outlined in [15]. The free-text comments were reviewed by 2 independent researchers, who identified overarching categories through a consensus process. The comments were then categorized and quantified, with a representative example selected for each category.

### Ethical Considerations

#### Human Subject Ethics Review Approval

The local institutional review and ethics board (Medical Ethics Committee at the University of Würzburg) judged the project as not representing medical or epidemiological research on human subjects and as such adopted a simplified assessment protocol. The project was approved without any reservation under the proposal number 20230323-03.

#### Informed Consent

Students were informed about the study, and their participation was voluntary. Written informed consent was obtained from all participants, who were also provided with information on data processing for the analysis and the publication of results. Contact details were supplied for participants wishing to withdraw their consent to data processing. The decision to participate or not had no consequences on the students’ academic progress.

#### Privacy and Confidentiality

Survey data from the questionnaires were collected anonymously using the EvaSys platform (Evasys GmbH, Lüneburg, Germany). Data were processed and stored in accordance with local data protection laws.

#### Compensation Details

No compensation was provided to the participants.

## Results

### Student Participation and Feasibility Evaluation

**Figure 2:**
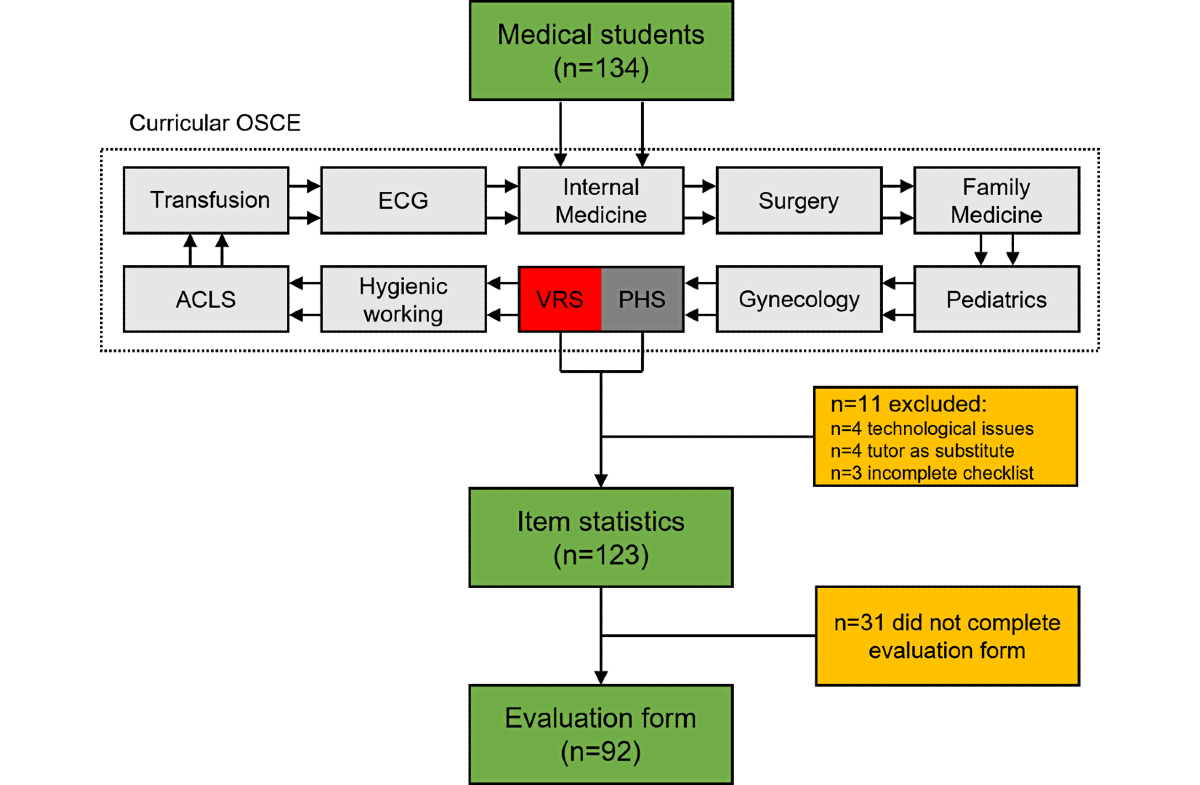
Layout of the objective structured clinical examination (OSCE) and data collection methods (green) used in the study from both the virtual reality–based station (VRS) and physical station (PHS). The number of participants, along with those excluded (yellow), is indicated. ACLS: advanced cardiac life support; ECG: electrocardiogram.

In total, 134 students participated in the OSCE examination. We excluded 11 students from the final analysis for various reasons: technical problems with the VR equipment (n=4), VR procedures executed by a tutor instead of the student (n=4), and incomplete assessment forms (n=3). Thus, 123 participants completed either the VRS (n=57) or PHS (n = 66; Figure 2).

The feasibility evaluation (Multimedia Appendix 3) confirmed that the implementation of the VRS proceeded smoothly, without major organizational challenges. From a technical perspective, 53 of 57 students (93%) were able to use the VR technology without any issues. The transition to VR did not negatively impact the overall process nor performance of the OSCE, and the modular design of the OSCE stations facilitated seamless integration of the VRS. Regarding the cost comparison, approximately €750 (US $776.42) per semester was saved with the VRS than with the PHS, primarily due to lower expenses for SPs and consumables. However, these savings must be considered in light of the total development costs, which amounted to approximately €25,000 (US $25,880.50) for the 2 VR scenarios used.

### Performance Data and Item Characteristics

The characteristics of items across all stations are presented in Table 1. The overall average item difficulty P was 0.68. In VRS, septic shock and anaphylactic shock exhibited similar item difficulties at 0.67 and 0.58, respectively, which were comparable to the same scenarios in PHS (0.71 and 0.64, respectively). Additionally, the mean item difficulty for scenarios from the other 5 case-based stations consistently aligned at 0.71. Concerning item discrimination, the overall average was r’=0.30. The VRS scenarios proved to be above average with values of 0.40 (septic shock) and 0.33 (anaphylactic shock), in contrast to the PHS scenarios, for which below-average values of 0.12 and 0.25, respectively, were determined. The scenarios from surgery, pediatrics, and gynecology demonstrated values that were comparable or even better than those for the VRS, while others (internal medicine, family medicine) had significantly lower values. When calculating the discrimination index D, an average of 0.16 was observed across all stations. The VRS scenarios outperformed all other stations, achieving the highest values of 0.25 and 0.26. Conversely, the PHS scenarios fell below this average, with values of 0.10 and 0.12. The other case-based scenarios presented a diverse range of values, mirroring the subject-specific trends seen in item discrimination, with an average of around 0.15.

**Table 1:** Item characteristics of the objective structured clinical examination (OSCE) stations for the virtual reality–based station (VRS) and physical station (PHS) in the specific scenarios, as well as the other 5 case-based stations—each comprising 4 scenarios.

Scenarios		Item difficulty (P)	Item discrimination (r')	Discrimination index (D)
**VRS**				
	Sepsis (n=32)	0.67	0.40	0.25
	Anaphylaxis (n=25)	0.58	0.33	0.26
**PHS**				
	Sepsis (n=31)	0.71	0.12	0.10
	Anaphylaxis (n=35)	0.64	0.25	0.12
Internal medicine (n=134)		0.68	0.18	0.09
Surgery (n=134)		0.72	0.49	0.22
Family medicine (n=134)		0.78	0.12	0.08
Pediatrics (n=133)		0.67	0.44	0.18
Gynecology (n=134)		0.70	0.40	0.17
All stations		0.68^a^	0.30^a^	0.16^a^

^a^Mean value shown.

### Quantitative and Qualitative Results of Survey Data

The online survey was completed by 92 participants following the OSCE, resulting in a 74.8% (92/123) response rate. Table 2 depicts the gender distribution of the participants, as well as their previous experience with emergency medicine and VR. No notable disparities were identified between the VRS and PHS groups.

Students rated their perceptions and attitudes toward the VRS or PHS (Table 3). Moderate scores were recorded for the perception of stress, with no significant difference between the VRS and PHS. Usability was scored favorably, and both formats were viewed as effective in demonstrating acquired skills. Students felt they only had a moderate level of preparation from the general curriculum for the station they completed. However, the specific materials provided enhanced their readiness. Students from both the VRS and PHS groups found their respective scenario to be a realistic portrayal of medical emergencies and clinically relevant. They rated the scenarios as manageable within the given time. Students believed that the station allowed them to demonstrate their skills and felt they handled the station well. Overall, students found the OSCE worthwhile as an examination format in medical school. They perceived the type of examination station they completed as meaningful. Notably, compared with the VRS, there was a significant preference among students for increased use of the PHS in future assessments.

**Table 2:** Participant gender and experience with emergency medicine, as well as characteristics of their experience with virtual reality (VR) categorized by the total sample, those using the VR-based station (VRS), and those using the physical station (PHS).

Characteristics		Total (n=92), n (%)	VRS (n=43), n (%)	PHS (n=49), n (%)	*P* value^a^	
**Gender**						.86
	Male	39 (42)	18 (42)	21 (43)		
	Female	52 (57)	25 (85)	27 (55)		
	Diverse	1 (1)	0	1 (2)		
**Experience in emergency medicine (eg, volunteer service)?**						.74
	Yes	20 (22)	10 (23)	10 (20)		
	No	72 (78)	33 (77)	39 (80)		
**Cumulative experience with VR applications (hours)**						.29
	0	23 (25)	14 (33)	9 (18)		
	0-5	67 (73)	28 (65)	39 (80)		
	6-10	2 (2)	1 (2)	1 (2)		
	>10	0	0	0		
**Use of the VR lab for preparation**						.97
	Yes	28 (30)	13 (30)	15 (31)		
	No	64 (70)	30 (70)	34 (69)		

^a^Comparison between the VRS and PHS groups using the Wilcoxon rank-sum test.

**Table 3:** Students’ perceptions and attitudes toward the 2 specific objective structured clinical examination (OSCE) modalities (virtual reality–based station [VRS] and physical station [PHS]).

Theme and items^a^		VRS (n=43), mean (SD)	PHS (n=49), mean (SD)	*P* value^b^
**Stress**				
	1. I felt stressed because many aspects of the scenario were beyond my control.	2.28 (1.26)	2.51 (1.19)	.30
	2. I felt stressed because I lacked sufficient medical knowledge to handle the case.	2.56 (1.14)	2.39 (1.06)	.61
	3. The presence of the examination staff put pressure on me.	1.61 (0.98)	1.71 (1.17)	.87
**Usability**				
	4. The equipment worked without any issues.	3.63 (1.18)	3.74 (1.17)	.75
	5. I was able to perform the medical procedures as I had envisioned.	3.40 (1.20)	3.37 (1.24)	.92
**Preparation**				
	6. I felt adequately prepared for the station through the curriculum lectures and courses.	2.67 (1.06)	2.69 (1.23)	.87
	7. I felt adequately prepared for the station through the preparation materials.	3.23 (1.09)	3.61 (1.27)	.06
**Acceptance**				
	8. The scenario felt realistic.	3.21 (1.32)	3.25 (1.32)	.89
	9. I found the scenario manageable within the given time.	3.88 (1.12)	4.08 (0.89)	.54
	10. The content of the scenario was clinically relevant.	4.47 (0.59)	4.45 (0.82)	.68
	11. This type of station should be used more frequently as an examination format.	3.07 (1.20)	3.57 (1.40)	.04
	12. Overall, I would rate the station as:^c^	3.58 (1.10)	3.78 (1.10)	.32
**Performance**				
	13. The station provided me with an opportunity to demonstrate my learned skills.	3.61 (0.90)	3.61 (1.22)	.70
	14. I was able to handle the examination station well.	3.40 (0.88)	3.53 (1.00)	.35
	15. I would rate my performance on this station as:^c^	3.44 (0.80)	3.57 (0.84)	.40
**General rating**				
	16. I believe OSCE examinations in medical school are generally worthwhile.	3.49 (1.32)	3.55 (1.21)	.92
	17. I find the type of examination station I completed to be meaningful.	3.05 (1.27)	3.53 (1.24)	.07
	18. This type of station should continue to be a part of the OSCE examination.	3.07 (1.37)	3.65 (1.18)	.03

^a^Rated on a 5-point Likert scale ranging from 1 (strongly disagree) to 5 (strongly agree).

^b^Comparison between the VRS and PHS groups using the Wilcoxon rank-sum test.

^c^Rated on a 5-point Likert scale ranging from 1 (very poor) to 5 (very good).

We subsequently performed thematic analysis on the answers to the open-ended questions from the VRS participants to provide some context for the quantitative findings and to gain deeper insights into students’ perspectives on using VR technology in examinations. From the open-ended responses, we identified both positive and negative themes (Table 4) encompassing usability, difficulty or fairness, preparation, practical relevance, and general feedback. Of note, there were nearly twice as many positive (n=58) as negative comments (n=30). Students predominantly praised the realism and fairness of the examination, with approximately 35% (20/58) and 30% (17/58), respectively, of the positive comments focused on these aspects. These themes also emerged less frequently in a negative context, often from students who faced challenges with time constraints or found some interactions in VR as abstract. Difficulties with the technology were identified by 8 participants, who experienced minor issues or challenges. Other notable positive feedback included a generally positive outlook toward the VRS and a strong appreciation for the relevance of the medical content.

**Table 4:** Summary of positive and negative feedback on the virtual reality–based station (VRS) from the open-ended questions.

Theme		Responses, n (%)^a^	Example quote			
**Positive themes (n=58)**						
	Realism	20 (34)	“The setting was realistic and provided ample room for action.”			
	Fairness	17 (29)	“The station was manageable within the time frame. There was [technical] assistance available when needed.”			
	General	7 (12)	“I had fun during the examination.”			
	Relevance	6 (10)	“Responding in an emergency situation is a highly relevant content.”			
	Usability	5 (9)	“The operation was reliable and intuitive.”			
	Preparation	3 (5)	“The preparation served as good practice.”			
**Negative themes (n=30)**						
	Realism	9 (30)		“The medications and procedures could be made somewhat more realistic.”		
	Fairness	9 (30)		“I would have needed more time and more support.”		
	General	2 (7)		“The VR^b^ station should not be part of a graded examination.”		
	Relevance	0		N/A^c^		
	Usability	8 (27)		“The handling could be optimized. Additionally, I experienced technical disruptions, which could also be addressed.”		
	Preparation	2 (7)		“Better preparation through teaching is needed for medical emergencies.”		

^a^Multiple responses were allowed. The percentages refer to responses given in each specific category (positive or negative) and not to the total number of respondents.

^b^VR: virtual reality.

^c^N/A: not applicable.

## Discussion

### Principal Findings

In our study, we integrated a VRS featuring 2 complex emergency scenarios into the established OSCE for advanced medical students at our institution. We found that the VRS presented a comparable level of difficulty to the traditional PHS stations. Notably, the VRS was more effective in distinguishing between high and low-performing students, as demonstrated by higher item discrimination and discrimination index values. Many students, including first-time VR users, praised the VRS for its realistic portrayal of medical emergencies and the fair assessment conditions provided. However, some expressed hesitancy about the broader application of VR in future practical assessments.

The integration of the VRS into the existing OSCE framework proved feasible both technically and organizationally. This is evidenced by successful participation of 134 students in the OSCE, with 53 students (or their tutor representatives) completing the tasks on the VRS without substantial issues. Our feasibility evaluation suggests that VRS can be implemented both practically and efficiently, even within the constraints of tight examination schedules. Since this is the first description of a VRS being included in a curriculum-based OSCE for a full cohort, it is difficult to compare costs more broadly. A recent review discussed the expenses related to VR-based training, including scenario development costs [16]. Consistent with our findings and those of others [17], these initial development costs can be offset by substantial long-term savings in recurring expenses such as SPs and consumables. Furthermore, once developed, the VR scenarios can be reused in various contexts without additional costs.

Analysis of performance data revealed that the item statistics for the VRS were not only comparable to, but in some cases even superior to, those of the PHS and the physical case-based scenarios from the 5 medical disciplines. Such good item characteristics and high discrimination values align well with observations from other studies. Recently, the efficacy of VR-mediated 360° videos in differentiating various skill levels during assessments was highlighted [9]. Studies of telemedicine examinations during the COVID-19 era also suggest that the validity and reliability of digital examination tools can be strong [18,19]. Of note, the discrimination r' and discrimination index D of the VRS were superior to those of the PHS, despite the randomization of participants and rotation of examiners at the stations. One possible explanation for these findings is the more uniform setting offered by VR. This environment minimizes or even eliminates the variability [20] that might be introduced unintentionally by SPs, which can occur even when SPs are well-trained and experienced in their roles. Furthermore, the possibility exists that operating the VRS itself may constitute an additional task that high-performing students are more adept at handling, which could lead to greater differentiation in performance at these stations. In other words, there might be confounding variables (eg, spatial ability) related to achievement in VR environments [21], which could potentially enhance performance outcomes. This hypothesis warrants further investigation, such as examining the correlation between VR handling skills and overall academic performance. Such insights could prove valuable in the design of more complex digital examinations and case-based assessments.

The study clearly demonstrates that students generally responded positively to the VRS, indicating a favorable attitude and willingness to engage with and benefit from VR technology. The realism and content relevance of the VR scenarios received above-average ratings and were frequently commended in the qualitative feedback. Fair assessment conditions were emphasized, and even stress experienced during the examinations was considered manageable. This aligns with the findings of recent studies indicating that students exhibit a positive attitude [22] toward VR-based teaching and assessment. Furthermore, they perceive VR as engaging and immersive, affecting learning outcomes positively [9].

Presumably, owing to the lower level of technical refinement in its implementation, another study discovered that students were still more likely to accept VR in classroom settings as opposed to its use in practical assessments [23,24]. Of note, the VRS here was accepted by students across various levels of technological proficiency, including those with no prior VR experience. Interestingly, a substantial majority (64/92, 70%) of the students chose not to use the offers of extra preparation toward the VR examination. Moreover, for 25% (23/92) of the participants, this was their first ever experience with a VR application. These findings highlight the viability of VR as an examination tool, accommodating students with a wide range of familiarity with technology. This study did not concentrate on the viewpoint of assessors; another study has explored the feasibility and benefits of using specific VR scenarios in OSCEs, receiving positive evaluation from assessors [18].

Nevertheless, a degree of hesitancy among participants was recorded regarding whether VRS should be used in future examinations: Agreement with this statement was clearly less than that for the PHS. This aligns with findings from another study, which indicated that students’ reservations were primarily due to their lack of experience with VR technology [25]. The referenced study concluded that practical examinations using VR should only be considered once the technology is firmly established and has demonstrated reliability in educational contexts. Notably, some open-ended comments in our study expressed concerns regarding the potential increase in replacing human assessors and SPs with technology, aligning with findings from prior research [26]. A strategy to counteract this could involve clear communication that VR simulation is intended merely as an additional option to complement, not merely replace, existing examination formats. Reservation and reliance on technology during the early stages of implementation were also reasons why we still opted for manual recording during data collection, regardless of the fact that the VR software provided the capability of automated performance evaluation through a checklist. Nonetheless, the potential to use such automated features in the future to aid assessors in their demanding role is promising. This approach could represent a significant advancement in the efficiency and objectivity of future assessments, including approaches for formative feedback [27].

### Strengths of the Study

One advantage of the study is the use of hardware and software that have been undergoing continuous evaluation in learning contexts since 2018, providing a high level of realism with minimal simulation sickness. Students were provided enough practice opportunities in the VR environment beforehand to minimalize operational issues (as demonstrated by good usability results). Nevertheless, the technology needs further rigorous development and enhancement to avoid issues in the examination context. Another strength of this study is the relatively large number of participants drawn from an entire semester cohort, which should generally be viewed as representative of the entire medical student population, including those with critical perspectives. The study’s greatest strength, however, lies in the comparison of item statistics and questionnaires directly between the VRS and PHS. This approach allows for the separation of effects related to scenario content and those related to the modality, which can influence the overall acceptance of and performance within the examination. Additionally, comparing results with other medical disciplines in the same examination aids in assessing the overall performance of the students.

### Limitations of the Study

One clear limitation is the restriction to a single institutional site and medical discipline. This is especially relevant to practical training, for which curricula can vary greatly across different faculties, thus limiting study generalizability. Another weakness is the absence of inferential statistical analysis that would correlate performance data with survey results. This was a consequence of the voluntary and anonymous nature of the questionnaires. Making participation in the survey mandatory may have overwhelmed the students and potentially affected acceptance of the VRS. Nevertheless, this analysis should be conducted in the future to identify students facing specific challenges with the VRS.

### Conclusions

Our study successfully demonstrated that complex VR-based assessment scenarios can be integrated into an established OSCE. Compared with the similar PHS, the VRS exhibited a similar level of difficulty but showed more favorable discrimination metrics. Student acceptance was high, with no major significant differences between VR-based and physical OSCE stations. Consequently, we encountered no systematic issues that would hinder the broader adoption of VR in assessing clinical competence.

## Appendix

Multimedia Appendix 1 Candidate assessment form items.

Multimedia Appendix 2 CONSORT-eHEALTH checklist v1.6.1.

Multimedia Appendix 3 Feasibility evaluation and cost comparison.

Multimedia Appendix 4 Raw performance data.

Multimedia Appendix 5 Raw survey data.

## Abbreviations

OSCE: objective structured clinical examination
SP: standardized patient

## Data Availability

The data sets generated during and analyzed during this study are available in Multimedia Appendix 4 and Multimedia Appendix 5.
