# Supplementary material for: Comparing Virtual Reality–Based and Traditional Physical Objective Structured Clinical Examination (OSCE) Stations for Clinical Competency Assessments: Randomized Controlled Trial
Source: J Med Internet Res. 2025 Jan 10;27:e55066. doi: 10.2196/55066 (PMC11759906; doi:10.2196/55066)
Supplement: Multimedia Appendix 1 [file jmir_v27i1e55066_app1.docx]

**Table S1.** Candidate assessment form for actions that should be taken during the examination for the scenario “Septic shock”. Scoring for each item was either binary (criteria met or not met, corresponding to 1 or 0 points) or ternary (criteria fully met, partially met, or not met, corresponding to 2, 1, or 0 points). In calculating the total sum, all items were equally weighted and normalized to a maximum of 1 point.

| **Candidate assessment form for the station “septic shock”** | | | |
| --- | --- | --- | --- |
| **Item** | **Result** | | |
| **1 Monitoring/Diagnostics** | | | |
| 1.1 O_2_ monitoring attached | Yes |  | No |
| 1.2 Blood cultures taken before antibiotic therapy  *(partially met: only one pair or sample taken at the same puncture site)* | Fully met | Partially met | Not met |
| 1.3 Arterial blood gas taken | Yes |  | No |
| **2 Therapy and final diagnosis** | | | |
| 2.1 Rapid volume replacement initiated  *(partially met: volume too slow (<500ml/h))* | Fully met | Partially met | Not met |
| 2.2 Catecholamines (norepinephrine) administered if MAP < 65 mmHg under/after fluid replacement  *(partially met: catecholamine administration without naming a specific drug)* | Fully met | Partially met | Not met |
| 2.3 Empirical antibiotics administered (1^st^ choice: piperacillin/tazobactam, Meropenem)  *(partially met: antibiotic therapy with a 2^nd^ choice drug)* | Fully met | Partially met | Not met |
| 2.4 Measures performed in correct order with volume administration as primary measure | Yes |  | no |
| 2.5 Correct suspected diagnosis named: Sepsis / septic shock  *(partially met: imprecise naming, needs assistance)* | Fully met | Partially met | Not met |
| **3 Further measures and recommendations** | | | |
| 3.1 Surgical consultation requested | Yes |  | no |
| 3.2 Intensive care unit transfer requested | Yes |  | no |

**Table S2.** Candidate assessment form for actions that should be taken during the examination for the scenario “Anaphylactic shock”. Scoring for each item was either binary (criteria met or not met, corresponding to 1 or 0 points) or ternary (criteria fully met, partially met, or not met, corresponding to 2, 1, or 0 points). In calculating the total sum, all items were equally weighted and normalized to a maximum of 1 point.

| **Candidate assessment form for the station “anaphylactic shock”** | | | |
| --- | --- | --- | --- |
| **Item** | **Result** | | |
| **1 Monitoring/Diagnostics** | | | |
| 1.1 O_2_ saturation monitoring attached | Yes |  | No |
| **2 Therapy and final diagnosis** | | | |
| 2.1 Allergen exposure stopped  *(partially met: not as first measure)* | Fully met | Partially met | Not met |
| 2.2 Epinephrine administered (0.15 to 0.6 mg IM / 1 µg/kg body weight IV)  *(partially met: correct dosage not stated)* | Fully met | Partially met | Not met |
| 2.3 Oxygen administered (5-12 L via mask)  *(partially met: insufficient flow rate)* | Fully met | Partially met | Not met |
| 2.4 Volume therapy initiated (> 500 mL/h)  *(partially met: insufficient flow rate)* | Yes |  | no |
| 2.5 Salbutamol administered | Yes |  | no |
| 2.6 Antihistamine administered | Yes |  | no |
| 2.7 Corticosteroid administered | Yes |  | no |
| 2.8 Structured approach with prioritization of administration of epinephrine and oxygen over further medication | Yes |  | no |
| 2.9 Correct suspected diagnosis named: Anaphylaxis / anaphylactic shock  *(partially met: imprecise naming, needs assistance)* | Fully met | Partially met | Not met |
| **3 Further measures and recommendations** | | | |
| 3.1 Monitoring for 24h recommended | Yes |  | no |
| 3.2 Emergency kit prescribed, recurrence risk mentioned  *(partially met: only one aspect)* | Fully met | Partially met | Not met |
| 3.3 Referral for allergological assessment advised | Yes |  | no |
